# The Effects of Mycophenolate on the Formation of Granulation Tissue Post-operatively in Canine Tracheal Stent Patients (2014–2020)

**DOI:** 10.3389/fvets.2021.697513

**Published:** 2021-08-03

**Authors:** Kevin F. Barber, Catherine A. Loughin, Dominic J. Marino, Martin Lesser

**Affiliations:** ^1^Department of Surgery, Long Island Veterinary Specialists, Plainview, NY, United States; ^2^Biostatistics Unit, Feinstein Institute for Medical Research, Northwell Healthy, Great Neck, NY, United States

**Keywords:** canine tracheal collapse, tracheal stent, granulation tissue, mycophenolate, immunomodulatory

## Abstract

**Objectives:** To determine if mycophenolate mofetil reduces the incidence and severity of granulation tissue in-growth in canine tracheal stent patients.

**Study design:** Randomized clinical trial.

**Animals:** 111 dogs from the hospital population.

**Methods:** Client-owned dogs that received an endoluminal self-expanding tracheal stent for canine tracheal collapse between 2014 and 2020 were randomly assigned into one of two treatment groups. Control group medication protocol consisted of prednisone 0.5 mg/kg PO BID/SID/EOD × 30 days, hydrocodone 0.25 mg/kg PO TID × 30 days, and cefovecin 8 mg/kg SQ post-placement. Mycophenolate group medication protocol was identical to the control group medication protocol with the addition of mycophenolate mofetil 10 mg/kg PO BID × 30 days, SID for life. Recheck tracheoscopy was performed at 1, 3, and 6 months post-stent placement. Presence and severity of granulation tissue were determined by tracheoscopy and were recorded as a percentage of tracheal lumen obstruction by blinded evaluators (none present, <25%, >25–50%, and >50%).

**Results:** At none of the three time points was there a statistically significant difference in grade between controls and those receiving mycophenolate (*p* = 0.467, *p* = 0.330, and *p* = 0.410).

**Conclusions and Clinical Significance:** Our results suggest that mycophenolate can be safely given to these patients but do not support that its administration will reduce the incidence and severity of granulation tissue. Although a difference was observed in the severity of granulation tissue between the two groups, loss to follow-up may have influenced conclusions. A larger study would be warranted to further evaluate the effect of mycophenolate on the development of granulation tissue.

## Introduction

Canine tracheal collapse syndrome is a progressive condition characterized by the degeneration of tracheal cartilage rings, leading to life-threatening dynamic airway collapse in the most severe of cases, with no curative treatment available (1). Many dogs will respond to medical management including various pharmaceuticals (glucocorticoids, antitussives, sedatives, bronchodilators, and antibiotics) (1, 2). In the most severe cases, in which medical management has been exhausted and failed to control clinical signs, surgical intervention is often considered (3). The most common procedures reported for the management of tracheal collapse include the use of extraluminal ring prosthesis and intraluminal stent placement (1).

Intraluminal tracheal stent placement is a minimally invasive procedure with rapid post-operative improvement in most cases (4). Following stent placement, median survival time of 1,005 days has been reported with long-term clinical improvement in goose-honking or raspy breathing (89%) and dyspnea (84%) (5). Post-operative complications reported include stent fracture, granulation tissue in-growth within the tracheal lumen, pneumonia, tracheal infection, progressive tracheal collapse, and stent migration or shortening (5). Granulation tissue ingrowth is a common complication reported in 28 to 49% of dogs (1, 5). This inflammatory tissue leads to loss of tracheal lumen aperture and may progress to a life-threatening reduction in air flow.

The development of granulation tissue formation appears to be multifactorial, involving shearing forces at the stent–mucosal interface due to improper placement or tracheal malformation, secondary bacterial infection, the chemical composition of the stent, and molecular machinery behind inflammatory fibrosis (6). The formation of granulation tissue leads to failure of mucus clearance, atelectasis, airway obstruction, respiratory distress, and unacceptable loss of quality of life. Studies in human medicine have revealed immunosuppression given to lung transplant recipients, may have an inhibitory effect on granulation tissue formation in metallic airway stents (7).

Glucocorticoids are a mainstay in the treatment of immune-mediated diseases in veterinary medicine and are the first-line therapy following intraluminal tracheal stent placement (1). Prednisone or prednisolone at 0.5 mg/kg twice daily, tapering the dose gradually over weeks to months, is typically used in conjunction with antitussives, antibiotics, and bronchodilators, as needed (1, 4, 5, 8). Glucocorticoid monotherapy is efficacious in the majority of cases, but as previously stated, an unacceptable number of patients may still develop life-threatening granulation tissue ingrowth. The use of oral colchicine and doxycycline for treatment of granulation tissue following stent placement has been reported in a single case report, but there has been no further investigation into the use of other secondary immuno-modulatory agents (9). More recent studies focusing on methods to preserve long-term stent patency have led to the development of covered and drug-eluting stents as well as the *in vitro* and *in vivo* application of substances to suppress granulation tissue formation (6). Pharmaceuticals such as AKLK-5 inhibitor, benzamide, dexamethasone, mycophenolate, paclitaxel, proteinase inhibitors, and other anti-fibrotic/immunomodulatory drugs are currently being explored in the human field (10).

Mycophenolate mofetil is a lymphocytotoxic immunosuppressive drug used in human and companion animal medicine for the treatment of immune-mediated disease (11–23). Mycophenolate functions as an immunomodulatory agent by inhibiting inosine monophosphate dehydrogenase production by T and B lymphocytes after being hydrolyzed in the intestines to the parent compound, mycophenolic acid (16, 20–23). Mycophenolic acid thereby leads to inhibition in the synthesis of guanosine nucleotides, without which lymphocytes are unable to differentiate, proliferate, and produce immunoglobulins (16, 20, 22, 23). Studies have shown that mycophenolic acid can induce the apoptosis of activated T cells and inhibits B-cell lymphocytes and antibody formation (16, 20–23). In addition, mycophenolic acid has been shown *in vivo* to reduce the recruitment of monocytes into sites of graft rejection and inflammation and also increases apoptosis of monocytes (20). Previous studies in veterinary medicine have evaluated its use in myasthenia gravis, inflammatory bowel disease, immune-mediated hemolytic anemia, immune-mediated thrombocytopenia, and glomerulonephritis, with variable success rates (12, 14–18, 23).

The purpose of this randomized clinical trial was to evaluate the use of mycophenolate mofetil as an adjunctive immunosuppressive agent with glucocorticoids in client-owned dogs following intraluminal tracheal stent placement for canine tracheal collapse. We hypothesized that a treatment protocol using mycophenolate mofetil as an adjunctive agent with glucocorticoids would be well-tolerated, decrease the incidence, and reduce the severity of granulation tissue formation in intraluminal tracheal stent patients.

## Materials and Methods

### Case Selection

Dogs diagnosed with tracheal collapse between June 2014 and June 2020 by utilizing thoracic radiographs and tracheoscopy followed by tracheal stent placement under fluoroscopy were included in this prospective study. Tracheal stents were placed when dogs had failed initial medical management.

### Tracheoscopy, Radiographic Measurements, and Tracheal Stent Placement

Patients were pre-medicated with butorphanol 0.2 mg/kg IV, induced with propofol 4–10 mg/kg IV (to effect). Intermittent propofol bolus at 0.1 mg/kg IV was administered throughout the procedure to aid in airway evaluation and to maintain an adequate plane of anesthesia. In all patients, a specialized bronchoscopic adaptor and scavenging system were attached to the endotracheal tube. Oxygenation was provided by non-rebreathing circuit. Constant ECG, blood pressure, and pulse oximetry were monitored throughout the procedure.

Tracheoscopy was performed with 2.7-mm, 30° scope to confirm and grade tracheal collapse (Karl Storz, Tuttlingen, Germany). In each dog, the grade and extent of tracheal collapse were recorded according to previously defined scheme based on the percent reduction of luminal dimension (1).

Tracheal dimension measurements and stent placement were performed as previously described (4, 5). Left lateral, right lateral, and dorsoventral thoracic and right lateral cervical radiographs were obtained in anesthetized dogs using a commercially available digital radiographic system (E-Film, Los Angeles, CA, USA). The dog was placed in lateral recumbency, its neck is flexed ventrally to straighten the trachea, and the endotracheal tube cuff is inflated directly caudal to the cricoid cartilage. A hydrophilic guide wire (Weasel Wire; Infiniti Medical, Menlo Park, CA, USA) within a marker catheter (measuring catheter; Infiniti Medical, Menlo Park, CA, USA) was placed in the esophagus so that the marker catheter spanned the length of the trachea. Radiographic measurements were calibrated using the marker catheter. Positive pressure ventilation of 20 cm H_2_O was applied to expand the trachea to its maximum dimensions. A radiograph was taken to measure tracheal dimensions, including diameter and length. Maximal tracheal diameters were measured in the cervical, thoracic inlet, and intrathoracic regions. A tracheal stent with a nominal diameter (resting, fully expanded) ~2–3 mm larger than the largest tracheal diameter measured at positive pressure ventilation was selected. Measurements were performed based on right lateral cervical radiographs by one or both of the authors (DM and CL). As in previous studies and per manufacturer guidelines, oversizing the stent is required to prevent migration and decrease gaps between the stent and tracheal wall so as to encourage epithelialization and minimize mucus accumulation (4). With the use of a stent-shortening chart provided by the manufacturer (Infiniti Medical, Menlo Park, CA, USA), tracheal stent length was selected with the goal of the deployed stent extending approximately 10 mm caudal to the cricoid cartilage to 10 mm cranial to the carina. With the use of fluoroscopic guidance, the Nitinol self-expanding metallic stent (Vet Stent-Trachea, Infiniti Medical, Menlo Park, CA, USA) was placed as previously described (4, 5). Immediately post-stent placement, patients were carefully reintubated, and the endotracheal tube cuff was segmentally inflated under fluoroscopic guidance throughout the entire length of the stent to ensure maximal stent expansion. Following stent placement, repeat tracheoscopy was performed (2.7 mm, 30° scope) to assess for gap between stent and tracheal wall. As described in previous studies, if stent-wall gaps were noted during post-stent tracheoscopy, an endotracheal tube cuff was inflated within an incompletely expanded stent to achieve more extensive wall contact (4).

### Treatment Groups

Eligible patients were sequentially randomized, with allocations blinded to the investigators, which consisted of a control group and a mycophenolate group. The control medication protocol consisted of prednisone 0.5 mg/kg PO BID/SID/EOD × 30 days, hydrocodone 0.25 mg/kg PO TID × 30 days, and cefovecin 8 mg/kg SQ post-placement. Mycophenolate group medication protocol was identical to the control group medication protocol with the addition of mycophenolate mofetil 10 mg/kg PO BID × 30 days, SID indefinitely. The owners of the animals were not blind to the treatment arm that they were assigned to. Assessment of compliance was ensured through being the single source of medications to owners, with non-blinded support staff ensuring appropriate refills.

### Follow-Up Evaluation

Information collection was based on recheck tracheoscopy at the described intervals. Data recorded included signalment, weight, current medications, tracheal collapse grade, and age at first stenting procedure; tracheal stent diameter, length, and type; time to follow-up examination; stent complications; and cause of death. Granulation tissue was evaluated at three time points: 1, 3, and 6 months following stent placement. The timing of grade assessment was allowed to be offset by ±2 weeks. The presence and severity of granulation tissue were determined by recheck tracheoscopy performed (2.7 mm, 30° scope) by one of the two authors (DM and CL) who were blinded to the patient's treatment group. The percentage of tracheal lumen obstruction was subjectively estimated and given an ordinal score by the clinician (none, 1, <25% lumen aperture occlusion; 2, 25–50%; and 3, >50%).

### Exclusion Criteria

Reasons for exclusion from study analysis included patients who died or were euthanized due to progression of respiratory disease (DOD), patients who died or were euthanized for other reasons (DOR), patients lost to follow-up (LTF), and patients who could not be re-evaluated due to lack of owner compliance (LOC) with study protocol including repeat tracheoscopy and medication administration.

### Statistical Analysis

Proportional distributions of nominal and categorical variables were compared with the chi-square test of independence, unless more than 20% of expected frequencies were <5 and then Fisher's exact test was used. The non-parametric Wilcoxon rank sum test was used to compare the frequency of granulation tissue grades between the two groups at each of the three time points. All statistical analyses were performed using commercially and publicly available statistical software (SAS version 9.4; SAS Institute, Cary, NC, USA). Descriptive statistics are presented for baseline variables (age and weight).

## Results

### Population, Stent Sizes, Age, and Weight

During the study period, 111 dogs were eligible for inclusion and randomly assigned to the control or mycophenolate treatment groups: 83 dogs were in the control group and 28 in the mycophenolate group. In the control group were 45 neutered males, four intact males, 33 spayed females, and one intact female. Of 28 mycophenolate dogs, there were 20 neutered males and eight intact females. Yorkshire terriers were the most common breed affected, followed by Maltese, Pomeranians, Chihuahuas, Toy poodles, and Pugs (Table 1). In the control group, the average age was 8.95 years, and weight was 4.79 kg. In the mycophenolate group, the average age was 8.43 years, and weight was 4.56 kg (Table 2). Of the control group, 36, 33, and 33% of patients were available for analysis at the 1-, 3-, and 6-month time points, respectively. Of the mycophenolate group, 61, 64, and 17% of patients were available for analysis at the 1-, 3-, and 6-month time points, respectively (Table 3).

**Table 1:** Breed distribution of treatment groups.

**Breed**	**Control group # (%)**	**Mycophenolate group # (%)**	**Total**
Chihuahua	5 (6.02)	1 (3.57)	6
Coton de tulear	0 (0.00)	1 (3.57)	1
English bulldog	2 (2.41)	0 (0.00)	2
Fox terrier	1 (1.20)	0 (0.00)	1
Jack russell terrier	1 (1.20)	0 (0.00)	1
Jack russell terrier mix	1 (1.20)	0 (0.00)	1
Lhasa apso	0 (0.00)	1 (3.57)	1
Maltese	15 (18.07)	4 (14.29)	19
Morkie	1 (1.20)	1 (3.57)	2
Papillon	1 (1.20)	1 (3.57)	2
Pomeranian	13 (15.66)	3 (10.71)	16
Poodle	1 (1.20)	0 (0.00)	1
Pug	4 (4.82)	0 (0.00)	4
Shih tzu	1 (1.20)	0 (0.00)	1
Silky terrier	0 (0.00)	1 (3.57)	1
Toy fox terrier	0 (0.00)	1 (3.57)	1
Toy poodle	4 (4.82)	1 (3.57)	5
Yorkshire terrier	33 (39.76)	13 (46.43)	46
Total	83	28	111

**Table 2:** Age (years) and weight (kilograms) distribution of treatment groups.

**Treatment group**	***N***	**Variable**	**Mean ± Std Dev**	**Median (IQR)**
Control	83	Age	8.95 ± 3.36	9.00 (7.00–11.00)
		Weight	4.79 ± 3.03	4.04 (3.10–5.40)
Mycophenolate	28	Age	8.43 ± 3.14	8.50 (6.00–10.50)
		Weight	4.56 ± 1.49	4.71 (3.67–5.54)

**Table 3:** Outcome classification at 1, 3, and 6 months including due to death from respiratory disease (DOD), death for any other reason (DOR), loss to follow-up (LTF), and lack of owner compliance (LOC).

**Control group (*N* = 83)**	**Classification of patient for analysis at recheck intervals**	**1 month**	**3 months**	**6 months**
	Available for analysis	30 (36%)	28 (33%)	28 (33%)
	DOD	4 (5%)	6 (7%)	10 (12%)
	DOR	13 (16%)	18 (22%)	21 (25%)
	LTF	24 (29%)	17 (21%)	9 (11%)
	LOC	12 (15%)	14 (17%)	15 (18%)
**Mycophenolate group (** ***N*** **=** **28)**		**1 month**	**3 months**	**6 months**
	Available for Analysis	17 (61%)	18 (64%)	17 (61%)
	DOD	2 (7%)	2 (7%)	2 (7%)
	DOR	4 (14%)	4 (14%)	5 (18%)
	LTF	4 (14%)	3 (11%)	2 (7%)
	LOC	1 (4%)	1 (4%)	2 (7%)

### Granulation Tissue at 1 Month

Of the 111 dogs, 30 of 83 (36.1%) in the control group and 17 of 28 (60.7%) in the mycophenolate group returned for recheck tracheoscopy within the 1 month ± 2 week window (*p* = 0.023). In the control group, nine of 30 dogs had granulation tissue present (30.0%) with an average granulation tissue grade of 0.60 ± 1.07, whereas in the mycophenolate group, four of 17 dogs had granulation tissue present (23.53%) with an average granulation tissue grade of 0.24 ± 0.44 (Tables 4, 7).

**Table 4:** Presence and severity of granulation tissue at 1 month ± 2 week time point.

**Granulation tissue grade**	**Control group # (% of grade)**	**Mycophenolate group # (% of grade)**	**Total**
0	21 (61.8)	13 (38.2)	34
1	4 (50.0)	4 (50.0)	8
2	1 (100)	0 (0)	1
3	4 (100)	0 (0)	4
	30 (63.8)	17 (36.2)	47

In the control group, one dog had grade 1 granulation tissue (13.3%), one dog had grade 2 granulation tissue (3.3%), and four dogs had grade 3 granulation tissue present (13.3%) (Table 4). In the mycophenolate group, four dogs had grade 1 granulation tissue present (23.5%), and no dogs had grade 2 or 3 (0%) (Table 4).

### Granulation Tissue at 3 Months

Of the 111 dogs, 28 dogs (33.7%) in the control group and 18 (64.3%) in the mycophenolate group returned for recheck tracheoscopy at the 3 month ± 2 week window (*p* = 0.005). In the control group, 14 of 28 dogs had granulation tissue present (50.0%) with an average granulation tissue grade of 1.00 ± 1.22, whereas in the mycophenolate group, seven of 18 dogs had granulation tissue present (38.8%) with an average granulation tissue grade of 0.61 ± 0.92 (Tables 5, 7). In the control group, six dogs had grade 1 granulation tissue (21.4%), two dogs had grade 2 granulation tissue (7.1%), and six dogs had grade 3 granulation tissue present (21.4%) (Table 5). In the mycophenolate group, four dogs had grade 1 granulation tissue (22.2%), two dogs had grade 2 granulation tissue (11.1%), and one dog had grade 3 granulation tissue present (5.5%) (Table 5).

**Table 5:** Presence and severity of granulation tissue at 3 month ± 2 week time point.

**Granulation tissue grade**	**Control group # (% of grade)**	**Mycophenolate group # (% of grade)**	**Total**
0	14 (56.0)	11 (44.0)	25
1	6 (60.0)	4 (40.0)	10
2	2 (50.0)	2 (50.0)	4
3	6 (85.7)	1 (14.3)	7
	28 (60.9)	18 (39.1)	46

### Granulation Tissue at 6 Months

At the 6 month ± 2 week window, 28 dogs (33.7%) in the control group and 17 (60.7%) in the mycophenolate group returned for recheck tracheoscopy (*p* = 0.012). In the control group, 15 of 28 dogs had granulation tissue present (53.5%) with an average granulation tissue grade of 1.14 ± 1.27, whereas in the mycophenolate group, eight of 17 dogs had granulation tissue present (47.0%) with an average granulation tissue grade of 0.76 ± 1.03 (Tables 6, 7). In the control group, five dogs had grade 1 granulation tissue (17.86%), three dogs had grade 2 granulation tissue (10.7%), and seven dogs had grade 3 granulation tissue present (25.0%) (Table 6). In the mycophenolate group, five dogs had grade 1 granulation tissue (29.4%), one dog had grade 2 granulation tissue (5.8%), and two dogs had grade 3 granulation tissue present (11.7%) (Table 6).

**Table 6:** Presence and severity of granulation tissue at 6 month ± 2 week time point.

**Granulation tissue grade**	**Control group # (% of grade)**	**Mycophenolate group # (% of grade)**	**Total**
0	13 (59.1)	9 (40.9)	22
1	5 (50.0)	5 (50.0)	10
2	3 (75.0)	1 (25.0)	4
3	7 (77.8)	2 (22.2)	9
	28 (62.2)	17 (37.8)	45

At none of the three time points was there a statistically significant difference in grade between controls and those receiving mycophenolate (p = 0.467, p = 0.330, and p = 0.410) (Table 7).

**Table 7:** Granulation tissue grades summarized as median with interquartile range (IQR).

**Group**	**Time point**	**N**	**Median (IQR)**
Control	1 month	30	0.00 (0.00–1.00)
	3 months	28	0.50 (0.00–2.00)
	6 months	28	1.00 (0.00–2.50)
Mycophenolate	1 month	17	0.00 (0.00–0.00)
	3 months	18	0.00 (0.00–1.00)
	6 months	17	0.00 (0.00–1.00)

### Follow-Up

Within the control group, 40% (12/30) returned for recheck tracheoscopy at all time points (1, 3, and 6 months), 27% (8/30) were only available at 1 month, 20% (6/30) returned at 1 and 3 months, and 13% (4/30) returned at 1 and 6 months. Of the mycophenolate group, 65% (11/17) returned for recheck tracheoscopy at all time points, 17% (3/17) were only available at 1 month, 12% (2/17) returned at 1 and 3 months, and 6% (1/17) returned at 1 and 6 months.

## Discussion

To our knowledge, this is the first report of the use of mycophenolate for the attempted prevention/modification of granulation tissue formation in canine tracheal stent patients. Our results suggest that mycophenolate can be safely administered to these patients but do not support the hypothesis that the administration of mycophenolate will reduce the incidence and severity of granulation tissue. In this study, sequential randomization without balance due to blinding of the investigators leads to a 3:1 skewing of control group to mycophenolate group. Our study is further limited by data excluded from analysis due to death from disease, death from unrelated causes, patient loss to follow-up, and lack of owner compliance. This loss of data frequently hampers veterinary studies, and overall exclusion from analysis in our study was comparable with that previously reported in tracheal stent patients (5). In the current study, patients were compared at the 1-, 3-, or 6-month time point independently, including data from patients who did not complete the follow-up protocol in its entirety. Although the exclusion of patients who died or did not adhere to treatment precludes performing the ideal analysis, to remove further patients would undermine any statistical comparison, as well as further limit conclusions that might have been made of the mycophenolate treatment.

The production of exuberant inflammatory tissue or granu-lation tissue is a known consequence of stent placement. Our study reported granulation tissue at an increased frequency as compared with previous studies (2, 5, 24). It is the only study thus far in which granulation tissue was specifically monitored for at regular intervals (1, 3, and 6 months) with a sensitive diagnostic (direct visualization via tracheoscopy). In other studies, only patients with worsening clinical signs would have undergone diagnostics, which led to the identification of granulation tissue, and these diagnostics were not consistently employed across other studies (1, 2, 5, 24). This methodology may have resulted in the underreporting of subclinical cases in the aforementioned studies. With regard to the severity of granulation tissue seen, although there is an observational difference between the two groups, due to loss of follow-up, one cannot suggest significance. Despite lack of significance, the results of this study are a promising first step into employing a standardized monitoring protocol to clinically investigate the use of immunomodulatory drugs for the modification of granulation tissue formation in tracheal stent patients.

Adverse events directly related to mycophenolate administration were reported in one dog. In this case, it was determined that the owner accidentally administered 10 times the instructed dose for >1-month time period. The client reported a waxing–waning appetite, intermittent large volume diarrhea, and a dull hair coat with hypotrichosis. In this instance, following cessation of mycophenolate for 1 week and then resuming the medication at the prescribed dose, the patient's appetite normalized, and no further diarrhea was reported. These findings support the notion that mycophenolate can safely be used with appropriate monitoring and clinical follow-up in the majority of cases.

Tracheal malformation in addition to collapse is thought to pre-dispose affected patients to gaps or gutters even after stent placement, predisposing them to granulation tissue formation. However, a recent study revealed malformation type dogs with tissue ingrowth were not found to be at a significantly higher risk (5). These results suggest that the underlying pathophysiology behind granulation tissue development is not solely linked to gap formation between stent and tracheal wall. There is evidence that nickel-induced tissue inflammation occurs, suggesting that the mere presence of a bare metallic stent induces inflammatory fibrosis (25). Stent placement directly produces inflammation, and repetitive stent motion may cause trauma with mucosal inflammation, granulation tissue development, and fibrosis (6, 13). Shin et al. reported that expression of matrix metalloprotease-9 was significantly increase in tracheal granulation tissue formation induced by stent placement relative to levels found in normal tissue in rats. In a canine model, trauma alone was enough to induce granulation tissue, but bacterial contamination likely contributed as well (26).

In a recent study evaluating bacterial infection before and after stent placement, tracheal stent placement does not increase the overall rate of pathogenic bacterial infection in these patients (2). A limitation of this study was that after stent, cultures were only obtained in patients having complication such as stent fracture and tissue ingrowth, which is suspected to have increased the risk of obtaining a positive culture. The influence bacterial infection has on the development of granulation tissue has yet to be determined. Future studies harvesting cultures before and after stent placement could help determine if a correlation exist between bacterial infection and the severity of granulation tissue formation. In addition, although whether there is an effect of mycophenolate mofetil on bacterial infection in stent patients has yet to be established, the aforementioned study demonstrated no significant difference in the frequency of positive cultures obtained during steroid use before stent placement compared with cultures obtained after stent placement (2).

Our study had inherent limitations due to the imbalance of randomization, cases excluded from analysis, and a subjective grading system in assessing granulation tissue severity. Future studies should employ block randomization and further incentivization to improve return rates. Thus, far, there has been no standardized objective method for evaluating granulation tissue, and our study is the first to attempt to assess severity of granulation tissue in tracheal stent patients. Further studies should compare CT and tracheoscopy for long-term post-operative monitoring in tracheal stent patients. A combined approach coupled with image analysis software could be used for more objective analysis. Future studies should investigate pre and post-stent as well as repeat airway cultures during recheck tracheoscopy at 1, 3, and 6 months. While the current study may not demonstrate a statistically significant difference in patients receiving mycophenolate, it suggests that this medication can be safely used in these patients and serves as a model for further research into immunomodulatory drugs in canine tracheal stent placements. Continued research is necessary to determine more accurate stent placement, to refine stent chemical composition, to ascertain the role of bacterial infection, and to evaluate immunomodulatory as well as targeted molecular therapy to reduce the occurrence of life-threatening granulation tissue ingrowth.

## Data Availability Statement

The raw data supporting the conclusions of this article will be made available by the authors, without undue reservation.

## Ethics Statement

Written informed consent was obtained from the owners for the participation of their animals in this study. Ethical review and approval was not required.

## Author Contributions

All authors listed have made a substantial, direct and intellectual contribution to the work, and approved it for publication.

## Conflict of Interest

The authors declare that the research was conducted in the absence of any commercial or financial relationships that could be construed as a potential conflict of interest.

## Publisher's Note

All claims expressed in this article are solely those of the authors and do not necessarily represent those of their affiliated organizations, or those of the publisher, the editors and the reviewers. Any product that may be evaluated in this article, or claim that may be made by its manufacturer, is not guaranteed or endorsed by the publisher.
